# Supplementary figures and images for: Protective effect of pre-existing natural immunity in a nonhuman primate reinfection model of congenital cytomegalovirus infection
Source: PLoS Pathog. 2023 Oct 5;19(10):e1011646. doi: 10.1371/journal.ppat.1011646 (PMC10553354; doi:10.1371/journal.ppat.1011646)

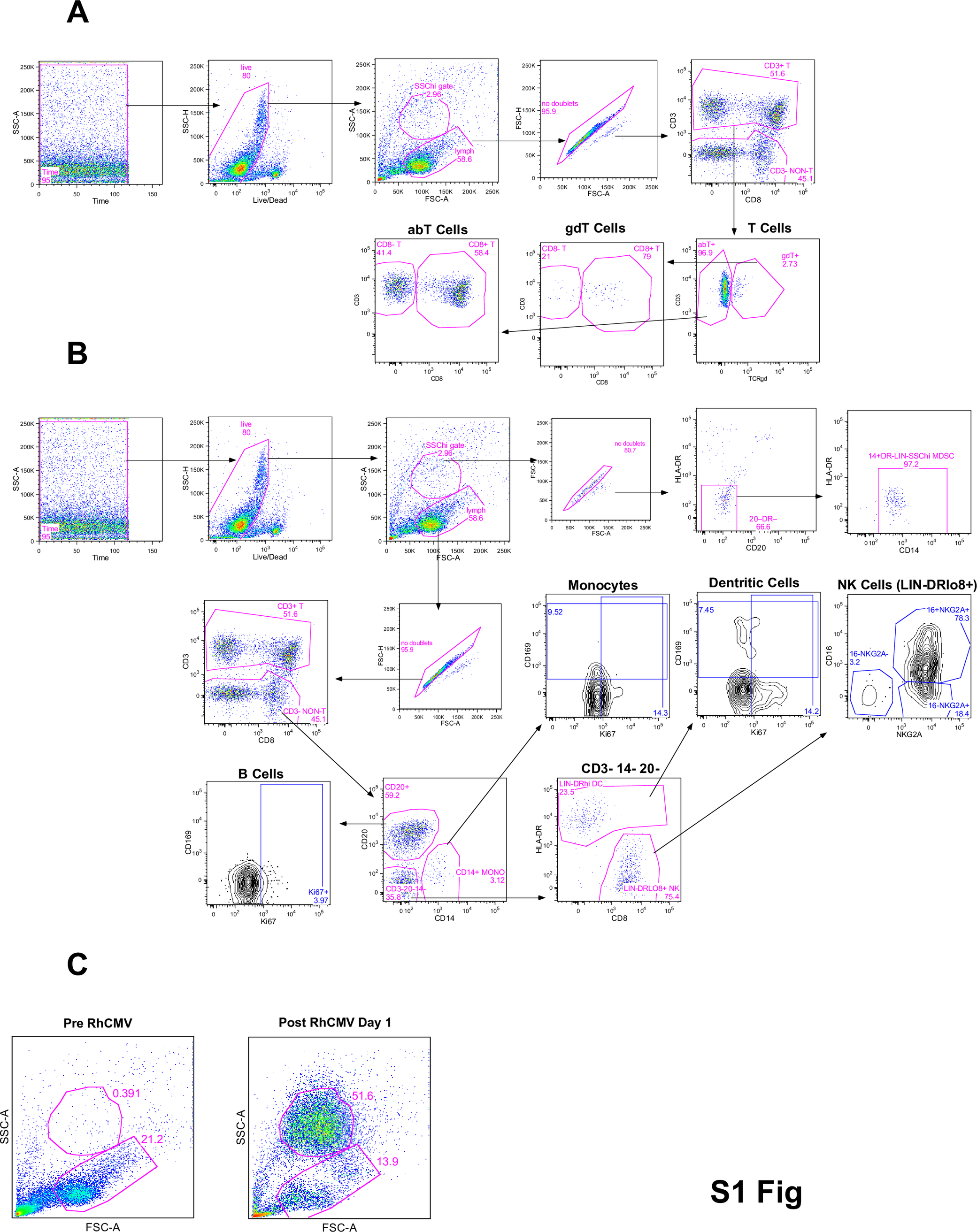

Supplement: S1 Fig — (A) Gating strategy used for the innate immune cell compartment. (B) The gating strategy used to identify T cell subsets. (C) Representative plots of side scatter high (SSChi) population following RhCMV reinfection in CMV-seropositive rhesus macaque dams. (TIF) [file ppat.1011646.s001.tif]

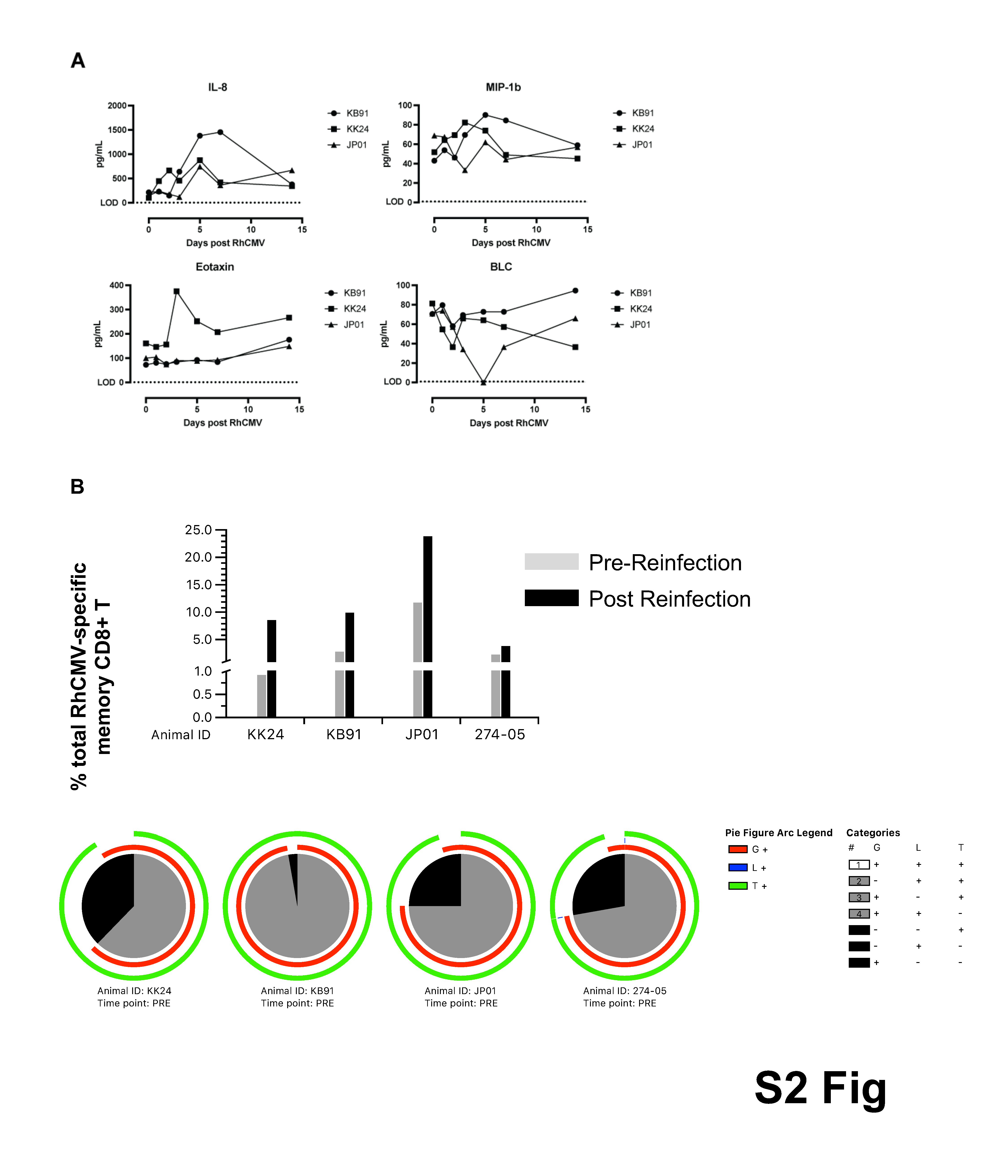

Supplement: S2 Fig — (A) Plasma IL-8, MIP-1b, Eotaxin, and B-lymphocyte chemoattractant (BLC) levels in three RhCMV reinfected dams in the first two weeks post RhCMV reinfection. Data generated using a nonhuman primate (NHP) 30-plex Luminex assay. Limit of detection (LOD) of the lot# is shown as a stippled line at the bottom of the y-axis. (B) Total memory RhCMV IE-specific CD8+ T lymphocyte responses at pre- and post reinfection time-points in four dams shown in the top panel. Post reinfection time-points varied between week 8 (KK24, KB91, JP01) and week 10 (274–05) post reinfection. Bottom panel showing pie charts depicting proportion of 3-functional, 2-functional and mono-functional RhCMV IE-specific CD8+ T lymphocyte responses prior to reinfection in the four dams. (TIF) [file ppat.1011646.s002.tif]

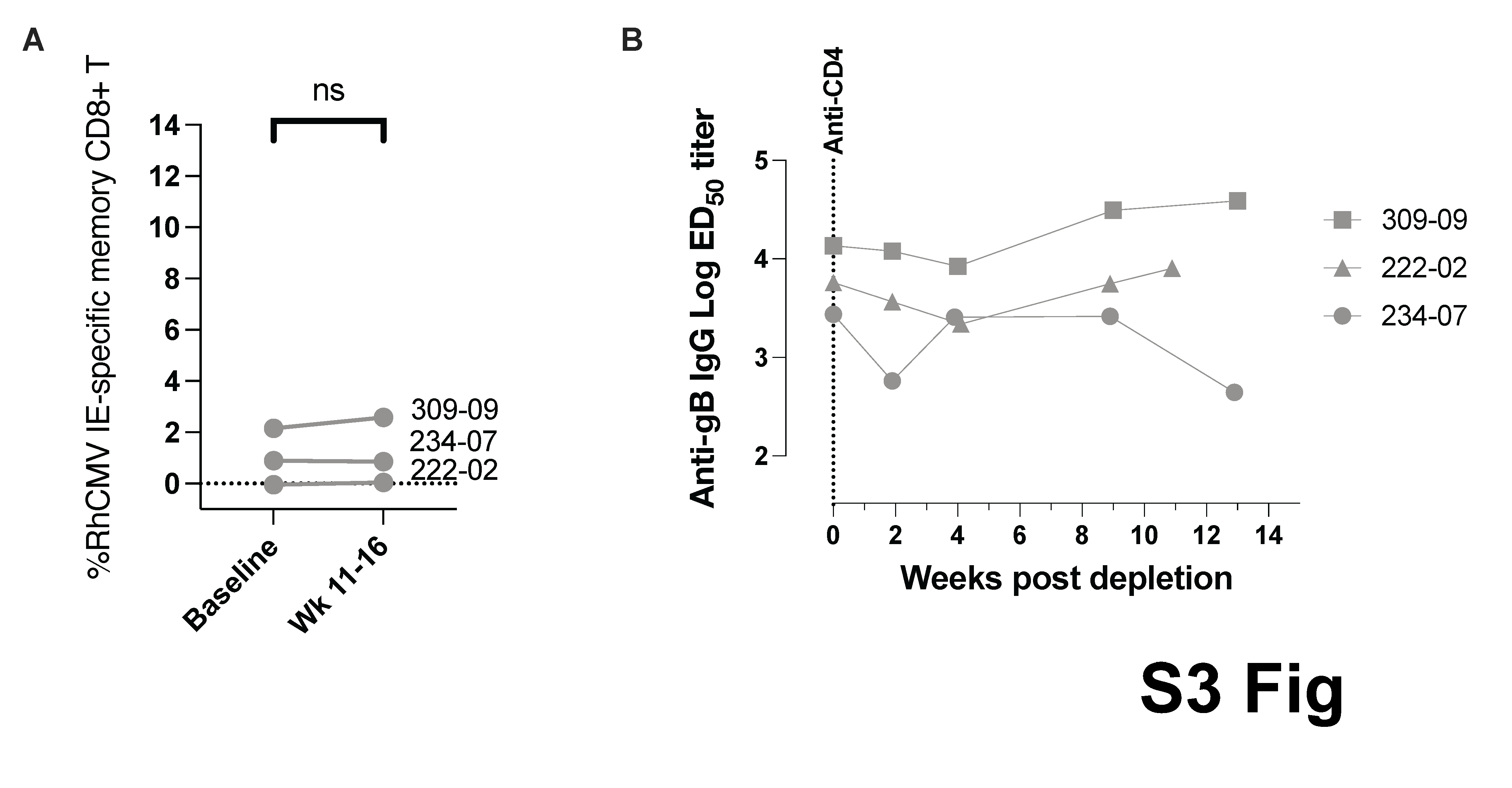

Supplement: S3 Fig — (A) Memory CD8+ T lymphocyte responses to RhCMV IE protein in CMV-seropositive controls. (B) Kinetics of RhCMV gB-specific binding antibodies in CMV-seropositive controls. (TIF) [file ppat.1011646.s003.tif]

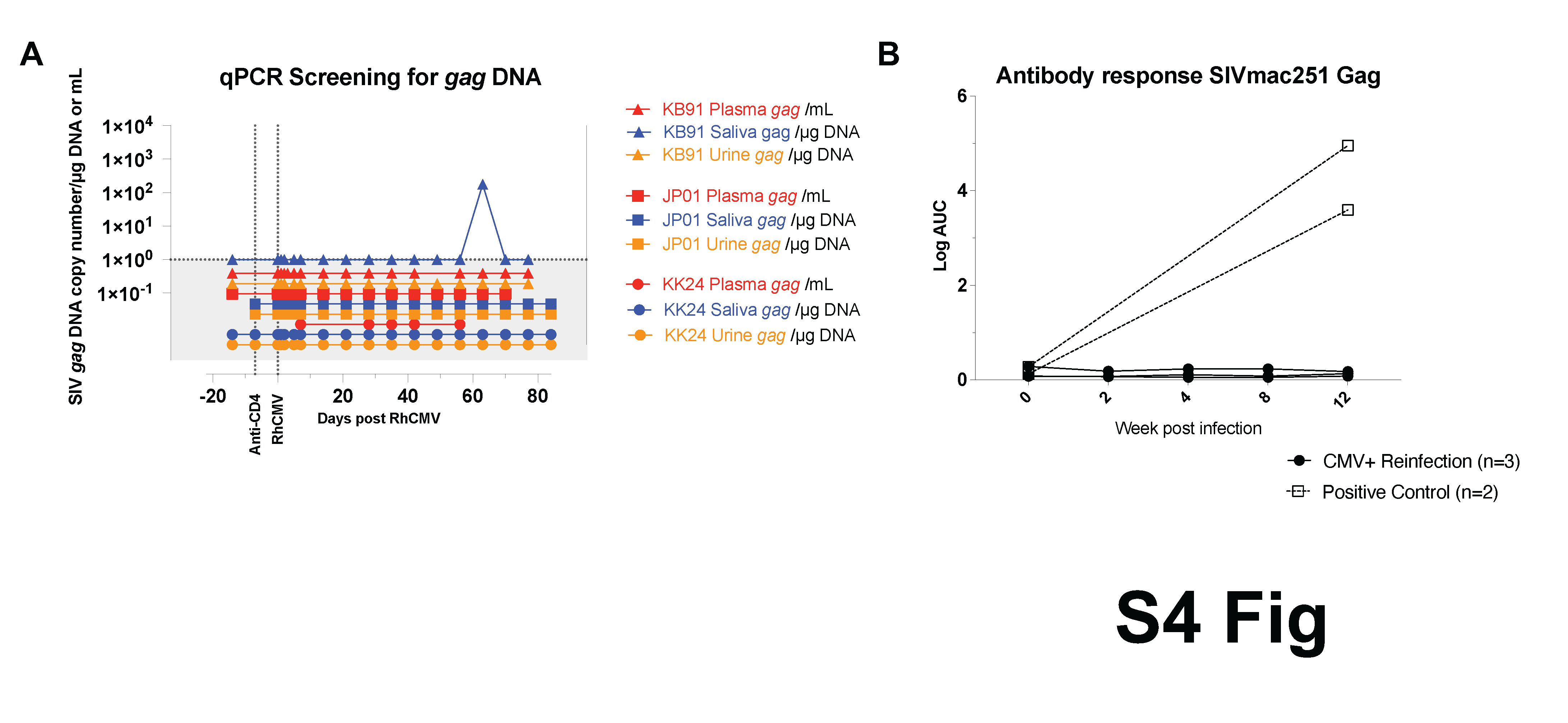

Supplement: S4 Fig — (A) Real time PCR for SIVgag DNA quantification performed on plasma, saliva and urine at multiple post reinfection time-points in three dams inoculated with FL-RhCMVΔRh13.1/SIVgag. Positive signal at a single time-point in KB91 saliva sample. (B) Gag-specific binding antibody assays in the three dams reinfected with FL-RhCMVΔRh13.1/SIVgag. Positive controls in this assay are SHIV-infected rhesus macaques (open symbol) were used for comparison of responses in CMV-seropositive reinfected animals (closed symbol). (TIF) [file ppat.1011646.s004.tif]

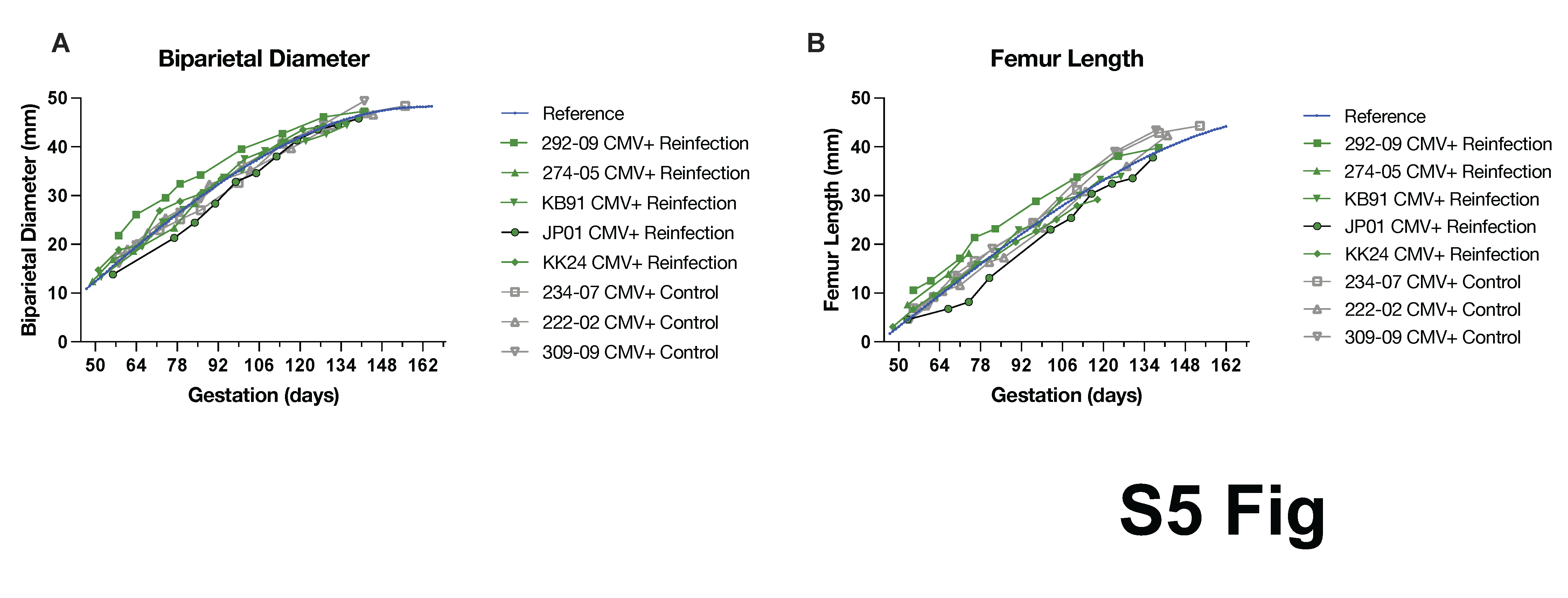

Supplement: S5 Fig — (A) BPD of CMV-seropositive reinfected (green) and CMV-seropositive controls (grey) fetuses compared to reference values [37]. (B) FL of CMV-seropositive reinfected (green) and CMV-seropositive control (grey) fetuses compared to reference values [37]. (TIF) [file ppat.1011646.s005.tif]
