# Supplementary material for: Protective effect of pre-existing natural immunity in a nonhuman primate reinfection model of congenital cytomegalovirus infection
Source: PLoS Pathog. 2023 Oct 5;19(10):e1011646. doi: 10.1371/journal.ppat.1011646 (PMC10553354; doi:10.1371/journal.ppat.1011646)
Supplement: S1 Table — (DOCX) [file ppat.1011646.s006.docx]

**S1 Table. Animal details for study groups outlined in Table 1.**

| **Study group** | **Animal ID** | **CMV seropositive / CD4 depletion** | **Date of CD4+ T depletion** | **Date of RhCMV inoculation** | **RhCMV strains used for infection (dose)** | **AF Tx** | **Previously reported** |
| --- | --- | --- | --- | --- | --- | --- | --- |
| CMV-seropositive Reinfection  (n=5) | 292-09 | Yes/Yes | 12/10/13 | 12/17/13 | **180.92**  (2x10^6^ TCID_50_) Intravenously in one arm | No | Current study |
|  | 274-05 | Yes/Yes | 02/04/14 | 02/11/14 |  | No |  |
|  | KB91 | Yes/Yes | 03/01/17 | 03/08/17 | **UCD52**  (1x10^6^ pfu) Intravenously in right saphenous vein  **FL-RhCMV/SIV*gag***  (1x10^6^ pfu) Intravenously in left saphenous vein | No |  |
|  | KK24 | Yes/Yes | 03/22/17 | 03/29/17 |  | Yes |  |
|  | JP01 | Yes/Yes | 05/10/17 | 05/17/17 |  | No |  |
| CMV-seropositive Controls  (n=3) | 234-07 | Yes/Yes | 08/14/13 | Not infected | Not infected | No | Group 3 in Bialas et al, PNAS 2015 |
|  | 222-02 | Yes/Yes | 12/04/13 | Not infected | Not infected | No |  |
|  | 309-09 | Yes/Yes | 12/10/13 | Not infected | Not infected | No |  |
| CMV-seronegative Primary infection  (n=6) | 145-97 | No/Yes | 04/11/13 | 04/18/13 | **180.92**  (2x10^6^ TCID_50_)  Intravenously in one arm  **UCD52***  (1x10^6^ pfu)  **UCD59***  (1x10^6^ pfu)  Intravenously in opposing arm in sequential syringes | Yes | Group 1 in Bialas et al, PNAS 2015 |
|  | 369-09 | No/Yes | 05/15/13 | 05/22/13 |  | Yes |  |
|  | 174-97 | No/Yes | 06/26/13 | 07/02/13 |  | Yes |  |
|  | 274-98 | No/Yes | 02/11/14 | 02/18/14 | **180.92**  (2x10^6^ TCID_50_) Intravenously in one arm | Yes |  |
|  | GM04 | No/Yes | 04/07/15 | 04/14/15 | **180.92**  (2x10^6^ TCID_50_) Intravenously in one arm  **UCD52***  (1x10^6^ pfu)  **UCD59***  (1x10^6^ pfu) Intravenously in opposing arm in sequential syringes | Yes | Nelson et al, JCI Insight 2017 |
|  | HD79 | No/Yes | 01/25/16 | 02/01/16 |  | Yes |  |

**AF Tx** – Amniotic Fluid transmission

*****previously reported as 1x10^6^ TCID_50_ rounded from 1.4 TCID_50_ (Bialas et al, PNAS 2015)
