# Supplementary material for: Protective effect of pre-existing natural immunity in a nonhuman primate reinfection model of congenital cytomegalovirus infection
Source: PLoS Pathog. 2023 Oct 5;19(10):e1011646. doi: 10.1371/journal.ppat.1011646 (PMC10553354; doi:10.1371/journal.ppat.1011646)
Supplement: S2 Table — (DOCX) [file ppat.1011646.s007.docx]

**S2 Table. RhCMV DNA PCR in placental and fetal tissues of CD4+ T lymphocyte-depleted dams.** NS = No sample. RhCMV DNA copy numbers shown as a heatmap; increasing values in shades of red. RhCMV DNA expressed as copies per μg input DNA in tissues and copies/ml in amniotic fluid.
