# Supplementary material for: Protective effect of pre-existing natural immunity in a nonhuman primate reinfection model of congenital cytomegalovirus infection
Source: PLoS Pathog. 2023 Oct 5;19(10):e1011646. doi: 10.1371/journal.ppat.1011646 (PMC10553354; doi:10.1371/journal.ppat.1011646)
Supplement: S3 Table — (DOCX) [file ppat.1011646.s008.docx]

**S3 Table. Antibodies for phenotyping and intracellular cytokine staining.**

| **Target** | **Fluorochrome** | **Company** | **Clone** | **Panel** | **Surface/Intracellular** |
| --- | --- | --- | --- | --- | --- |
| Live/Dead | BV510 | Invitrogen | Aqua | Phenotyping | Surface |
| CD3 | APC-Cy7 | BD | SP34-2 | Phenotyping/ICS | Surface |
| CD4 | PerCP-Cy5.5 | BD | L200 | ICS | Surface |
| CD8 | BV650 | BD | SK1 | Phenotyping/ICS | Surface |
| CD14 | BV605 | BD | M5E2 | Phenotyping | Surface |
| CD16 | BV711 | Biolegend | 3G8 | Phenotyping | Surface |
| CD20 | PacBlue | Biolegend | 2H7 | Phenotyping | Surface |
| CD28 | PE-CF594 | BD | CD28.2 | ICS | Surface |
| CD69 | APC | BioLegend | FN50 | ICS | Intracellular |
| CD95 | BV711 | BD | DX2 | ICS | Surface |
| CD107a | FITC | BD | H4A3 | ICS | Surface (1h into stimulation) |
| CD107b | FITC | BD | H4B4 | ICS | Surface (1h into stimulation) |
| CD169 | PE | Biolegend | 7-239 | Phenotyping | Surface |
| CD195 (CCR5) | PE | BD | 3A9 | ICS | Surface |
| Ki-67 | FITC | BD | B56 | Phenotyping | Intracellular |
| TCR γδ | PCP-Cy5.5 | BioLegend | B1 | Phenotyping | Surface |
| KIR2D | APC | Miltenyi | NKVFS1 | Phenotyping | Surface |
| Granzyme B | AL700 | BD | GB11 | Phenotyping | Intracellular |
| Granzyme B | BV421 | BD | GB11 | ICS | Intracellular |
| HLA-DR | PE-CF594 | BD | G46-6 | Phenotyping | Surface |
| NKG2A | PE-Cy7 | Beckman/Coulter | Z199 | Phenotyping | Surface |
| IFNγ | PE-Cy7 | BD | B27 | ICS | Intracellular |
| IL-2 | BV605 | BD | MQ1-17H12 | ICS | Intracellular |
| TNFα | AL700 | BD | Mab11 | ICS | Intracellular |
