# Supplementary material for: Protective effect of pre-existing natural immunity in a nonhuman primate reinfection model of congenital cytomegalovirus infection
Source: PLoS Pathog. 2023 Oct 5;19(10):e1011646. doi: 10.1371/journal.ppat.1011646 (PMC10553354; doi:10.1371/journal.ppat.1011646)
Supplement: Attachment — Submitted filename: Mostrom et al_Point-by-point response.v5.docx [file ppat.1011646.s010.docx]

We thank the reviewers for careful review of our manuscript and helpful comments. We appreciate the positive response and acknowledgement of the significance of the NHP model to investigate the role of pre-existing maternal immunity against cCMV.

The manuscript has been modified to address reviewer concerns. Modifications include text revisions / clarifications that are highlighted, correction of errors in Figures 1 and 5, a new supplementary figure (**Fig S4A**) with SIV gag PCR data on all animals, and a new supplementary table (**S1 Table**) listing the RhCMV strains and details of past animal experiments.

The point-by-point response to specific reviewer comments are listed below:

**Reviewer #1:**  There are a few minor concerns.

The group sizes of this study are small. Based on their results the authors suggest that CMV re-infection is more likely to contribute to placental transmission than reactivation. While none of the three control animals experienced placental transmission, only one of the five re-infected animals showed placental transmission, so it is unclear if there is a statistical difference.

- ***Response****: The reviewer is correct in pointing out the small sample size precludes definitive conclusions about contribution of reinfection vs reactivation in non-primary congenital CMV. The text (****lines 290-292 in results and lines 367-370 in discussion****) clarify the absence of statistical difference between the outcome in the CD4 depletion control and reinfection groups and acknowledges the small group sizes precludes definitive conclusions regarding the relative roles of reactivation and reinfection to non-primary cCMV.* ***Line 197-198 has been deleted****.*

The reinfection study also used a relatively high dose of viral inoculum (2 x 10E6), which could skew cCMV results towards re-infection rather than re-activation.

- ***Response:*** *The reviewer is correct. The text has been modified (****line 249-250****).*

Finally, it would be helpful for the authors to clarify or reconcile their data with observations that in the human population, roughly 50% are CMV sero-positive (which is hypothesized to be protective based on this study) and yet still accountable for roughly 50% of cCMV cases. These minor concerns do not diminish the value of this excellent work but some clarifications or discussions in the text will be helpful.

- ***Response****:* *The reviewer is correct that seropositive women account for the majority of the cCMV transmission globally. This epidemiology is due to two factors. First, the majority of women throughout the world are seropositive, with over 90% of women living in low and middle income countries being exposed to CMV prior to child-bearing years. Thus, even though there is a much lower chance of cCMV transmission after reinfection compared to new infection (30-40% vs 1-4%), the proportion of seropositive women is so high that this low rate of transmission still becomes the dominant transmission type.  Second, as seropositive individuals are more likely to cohabitate with other seropositive individuals, the rate of reinfection during pregnancy for a seropositive woman is up to 10 times higher than that of seronegative women (Permar, Schleiss, Plotkin review;* [*https://pubmed.ncbi.nlm.nih.gov/29343580/*](https://pubmed.ncbi.nlm.nih.gov/29343580/)*), thus the rate of exposure during pregnancy is also higher for seropositive women. Text modifications to elaborate on this point in* ***lines 69-73****).*

**Reviewer #2:** 
A variety of strains were used, including RhCMV strain 180.92 (n=2), a more wild-type strain of RhCMV that contains the homolog of UL128; RhCMV UCD52, and FL-RhCMVΔRh13.1/SIVgag, a wild type-like RhCMV clone with SIVgag inserted as an immunological marker (n=3). One thing I would like to see the others do is comment more about the genotype and phenotype correlations with 180.92 and UCD52. This has been published in the past, but in fairness to the reader the authors should plainly and simply state the differences in these strains, both genotypically and phenotypically, from past work. This reviewer would invite the authors to plainly and simply state which strain is more like a wild-type strain that would be encountered in a pregnant patient in the human context.

- ***Response****: The full-length RhCMV clone would be closest to an unpassaged wild-type strain that would be encountered in a pregnant patient.* ***Lines 143-152, and lines 161-164*** *added in text to address genotype and phenotype correlations for 180.92 and UCD52, and wild-type strain that best recapitulates human CMV exposure during pregnancy.*

The authors should also make it more clear (if indeed it’s true) that the experiment included a “mixing” experiment in which UCD52 was mixed with the FL-RhCMVΔRh13.1/SIVgag construct. This is implied in the abstract, but not stated clearly in the abstract. It should be stated with absolute clarity.

- ***Response****: We apologize for the confusion. This was not a “mixing” experiment. Each virus was administered separately on different arms. A clarification has been added to the abstract, results and methods (****line 31 in abstract; line 154 in results; line 465 in methods****).*

They conclude that reduced placental transmission and absence of fetal loss after non-primary as opposed to primary infection in CD4+ depleted dams indicates that preconception maternal CMV-specific CD8+ T lymphocyte and/or humoral immunity can protect against cCMV infection in this reinfection setting. There are two caveats to consider. One is the small number of monkeys studied. Even if reinfection leads to transmission associated-sequelae in human infants, the percentages are small. It would be an interesting exercise to calculate (although this reviewer doesn’t think the authors need to do this) to statistically surmise the “number of monkeys needed to treat” to see if sequelae were modified by pre-existing monkey immunity, if numbers were analogous to the human situation. What is the statistical power based on these small numbers?

- ***Response****:* *The rate of sequelae after cCMV infection due to maternal reinfection is <10% at birth in humans.  If the same biology played out in rhesus macaques, as only 1 in 5 reinfected dams transmitted the virus to their fetus, at least 50 dams would need to be reinfected to see 1 fetus with notable sequelae at birth.  Thus, a study of this size with pregnant rhesus macaques is not realistic.*

The second point circles back to the strain variation question. Transmission of two reinfection RhCMV strains across the placenta is noted, with ~30% corresponding to RhCMVΔRh13.1/SIVgag and ~70% to RhCMV UCD52. Again, please clarify is this was a “mixing” challenge study and state the rationale for “mixing” (which does not occur, at least not to this extent, in nature).

- ***Response****: This was not a “mixing” challenge experiment. Each virus was separately administered via the intravenous route on different arms of the animal. The identification of both inoculated variants in the amniotic fluid at variable proportions by sequencing is an indication that both viruses were able to cross the placenta and initiate an intrauterine infection. A clarification has been added to the abstract, results and methods (****line 31 in abstract; line 154 in results; line 465 in methods****) as described above.*

The inclusion of the SIVgag control is obvious, and useful (as noted above in this critique) but are there different levels of “fitness” for these viruses? Past work shows that UCD52 is wild-type in the pentameric complex region, in contrast to strain 68-1. But strain 180.92 is supposed to be UL128 positive, although it did undergo a lot of cell culture passage, both in rhesus and, interestingly, human cells (10.1128/JVI.80.8.4179-4182.2006). Page 7 states that the choices of inoculum strains were based on “past studies”. But the sequencing data does not mention any vertical transmission of 180.92 – only UCD52 and the derivative ΔRh13.1/SIVgag. Is this just because there was only two animals challenged (Table 1)? Or does UCD52 differ from 180.92 in terms of its transplacental transfer potential? If so, what is the molecular basis?

- ***Response****: RhCMV strain 180.92 was used in all the CD4-depletion primary infection experiments (Bialas et al 2015 PNAS and Nelson et al 2017 JCI Insight). When 180.92 was given along with RhCMV strains UCD52 and UCD59 (all separately administered in different IV lines), it was not detected or was present as a minor population. However, one CD4-depleted CMV-seronegative animal (dam 274-98 in Figure 1D in the Bialas et al paper and shown in new supplementary table S1) that received only 180.92 transmitted the virus across the placenta and spontaneously aborted within three weeks of RhCMV administration. Thus, 180.92 has transplacental transmission potential. This is not surprising as we have previously shown that the 180.92 infectious virus stock is a mixed virus; the dominant strain in the virus stock is the sequenced virus with a truncated UL/b’ region but intact UL128-UL131 locus. However, when administered in vivo, a minor wild-type like variant with a nontruncated UL/b’ region rapidly becomes the dominant strain in circulation and tissues (Assaf et al;* [*https://pubmed.ncbi.nlm.nih.gov/24899204/*](https://pubmed.ncbi.nlm.nih.gov/24899204/)*).* ***Lines 143-149*** *were added to describe 180.92.*

**Reviewer #3**: Overall, the study addressed a very important question in a NHP model that pre-existing immunity protects rhesus macaque dams from transmitting CMV to the fetus and from fetal loss even in the absence of CD4+ T cell response. However, there are few points that the authors should address before publication.

The major limitation of the study is the use of the historical data from a previous study in CMV-seronegative rhesus macaques. This would be somewhat offset if the exact challenge condition was used. However, the inoculum virus and dose were lower and maybe less pathogenic when compared to the condition used in the historical study:
1a. Historical study (Nelson et al. 2017 JCI Insights) used a mixture of virus stocks: 2e6 180.92+1e6 UCD52+1e6 UCD59 (all in TCID50 units). This study used 2e6 180.92 only, or 1e6 UCD52+1e6 FL-RhCMVΔRh13.1/SIVgag, thus the challenge dose is reduced by roughly 2-fold.

- ***Response****: We have included a new supplementary table (****S1 Table****) detailing virus inoculations in all the study groups outlined in Table 1. Although the numbers are small, it does not appear that two-fold differences in virus dose impacted the outcome. The historical controls used for the CD4-depletion primary CMV infection group included four dams from the Bialas et al study and two from the Nelson et al study (refer new S1 table); as pointed previously and shown in the* ***S1 Table****, one dam (274-98) from the Bialas et al study received only 180.92 at 2x10^6^ TCID_50_ and yet the virus transmitted across the placenta and led to RhCMV dissemination in the mother. The outcome in this dam was no different from the remaining five CD4-depleted dams that received two-fold more virus, i.e 2x10^6^ TCID_50_ of 180.92 plus 1x10^6^ TCID_50_ each of UCD52 and UCD59. A difference of two-fold TCID_50_ in virus titers could also be within the variability in titering assays. Finally it should be noted that in the reinfection experiments with UCD52 and* FL-RhCMVΔRh13.1/SIVgag *a dose of 1x10^6^ pfu of each virus was used (****S1 Table****). Assuming 1TCID_50_ is equivalent to 0.7 pfu, this would translate roughly to 1.4 TCID_50_ of each virus or roughly 3 TCID_50_ of total virus inoculum.*

1b. A separate historical study (Bialas et al. 2015 PNAS) has shown that inoculum of 180.92 alone resulted in more than 10-fold lower DNAemia than mixed viruses (180.92+UCD52+UCD59). In the latter group (challenge with mixed viruses), UCD52 was dominant among the three viruses in transmitting to fetus. Both data point to the direction that 180.92 is less pathogenic in terms of cCMV transmission, which was used for 2 out of 5 NHPs in the critical CMV-seropositive T cell depleted group in this study.

- ***Response:*** *The reviewer is correct about the lower plasma viral load with 180.92 alone. However, 180.92 when administered alone produced the same outcome as the combination of the three viruses (Bialas et al PNAS 2015).*

1c. Furthermore, 2 out of 5 NHPs in the critical CMV-seropositive T cell depleted group did not have an ideal depletion, when compared to the control group and the historical controls.

- ***Response:*** *The reviewer is correct about the incomplete peripheral blood CD4+ T lymphocyte deletion in two reinfected dams. Animal-to-animal variation in the extent of CD4+ T lymphocyte depletion after administration of the CD4 depleting antibody can occur (*[*https://www.nhpreagents.org/Portals/0/TDSFiles/Anti-CD4 [CD4R1] - PR-0407 NHP11 - TDS v2020.09.26.pdf?ver=2021-05-18-113935-040*](https://www.nhpreagents.org/Portals/0/TDSFiles/Anti-CD4%20%5bCD4R1%5d%20-%20PR-0407%20NHP11%20-%20TDS%20v2020.09.26.pdf?ver=2021-05-18-113935-040)*). Nevertheless, it is worth noting that the reinfected dam KK24 (Figure 1B) with the least effective CD4+ T lymphocyte depletion was the one that had evidence of placental transmission. This point was included in the discussion to highlight the impact of variable CD4+ T cell depletion efficacy.*

1d. Therefore, these three major differences on key experimental parameters may decrease confidence of the major conclusion of this study.

- ***Response:*** *A new supplementary table (****S1 Table****) lists out the animals and virus administered for each historical control and current study animals.*

**Part III – Minor Issues: Editorial and Data Presentation Modifications**
Please use this section for editorial suggestions as well as relatively minor modifications of existing data that would enhance clarity.

Reviewer #1: Fig 1A: It will be helpful if the authors can clarify the rationale for using both RhCMV strain 180.92 and UCD52 in this study, and also which animal was reinfected with which virus strain. The information is somewhere there but it will be better to have this at the beginning.

- ***Response****: This information was provided in the original text (****lines 155-157*** *of revised ms and in Figure 2 legend;* ***lines 763-765*** *of revised ms). The animal IDs have also been included on the results section of the revised text (see highlighted text in* ***lines 141,152,164****). We have added a supplementary table (****S1 Table****) listing all animals in this study and the historical controls used for comparison with primary infection*

Fig 1B: the labels of individual curves are confusing, particularly JP01 vs. 292-09, and 274-05 vs. KK24

- ***Response****: We apologize for the error. Figure 1B has been corrected.*

  Fig 2B: As stated above, the group sizes of this study are small (i.e. 1/5 of reinfected animals vs. 0/3 of control animals were transmitters) and the re-infection dose is high so that it is difficult to draw a firm conclusion as stated on Line 177: “Taken together, these data demonstrate placental transmission after RhCMV reinfection but not after reactivation”. The authors should clearly state the caveats of the study.
- ***Response****: The reviewer is correct. This sentence has been removed (see* ***line 197-198*** *of marked revised text). Caveats are discussed in* ***lines 290-292 in results and lines 367-370 in discussion.***

Fig 4B: Please clarify what the size of the pier chart stands for. For instance, in 4C, 3-functional T cells are shown as 10% but in 4B, it appears to be 75% of the pier chart.

- ***Response****: The bar chart in 4C is showing the actual frequency or magnitude of memory CD8+ T lymphocytes responding to RhCMV IE peptides and displaying all 4-functions, or 3-functions etc. The pie chart in 4B depicts the proportion of the total IE-specific response (irrespective of the frequency) with 4-, 3-, 2- or 1-function. Figure 4 legend has been modified for clarity (highlighted sections in* ***lines 778-786****)*

Fig 5A: It appears that the colors of CD4+ T cell and LogED50 curves are mislabeled for 274-05.

- ***Response****: We apologize for the error. Figure 5A has been corrected.*

  Fig 5C: please clarify if these are folds of the increase.
- ***Response****: These are change from baseline. The y-axis label has been changed for clarification and the legend modified (****lines 796-800****)*

Line 227: The authors stated that “our results demonstrate elevation of RhCMV-specific CD8+ T lymphocyte and antibody responses that was evident only after RhCMV reinfection, not reactivation following CD4+ T lymphocyte depletion”. Again one caveat here is that the inoculation dose is high, which could skew the result towards re-infection.

- ***Response:*** *The reviewer is correct.* ***Lines 197-198*** *has been deleted. A caveat line has been included (****lines 249-250*** *in revised ms)*

Line 247: The authors stated that “the resulting whole genome sequencing data (mean read length: ~200 bp) was mapped against the RhCMV UCD52 and FL-RhCMVΔRh13.1/SIVgag reference assemblies”. Please clarify if these reads are unique to these two viral strains.

- ***Response****: The majority of reads mapped equally well to either reference assembly. However, there are a few uniquely mapping regions that we were able to use to distinguish the two strains. From these regions, 31.1% correspond
  to RhCMV 68-1 (backbone for FL-RhCMVΔRh13.1/SIVgag* ) *and 68.9% to RhCMV UCD52 (though please keep in mind that we were working with consensus sequences and ignoring recombination). Clarification sentences added to revised text (****lines 269-271****).*

Reviewer #2: Consider expanding Table 1 to include columns describing in more details the differences, both genotypic and phenotypic, of strains 180.92 and UCD52.

- ***Response****: Text has been added to results section (****lines 143-152****) elaborating on 180.92 and UCD52.*

Reviewer #3: 2. Some key figure legends, text descriptions, and assay result lack consistency and technical rigor:
2a. Figure 1B: Two sets of two NHPs share the same label.

- ***Response****: Thank you for pointing out the error. Figure 1B labels have been revised.*

2b. Figure 2A: Which two NHPS received only 180.92?

- ***Response****: Dams 274-05 and 292-09 denoted in Figure 2 legend. Additional text in the results section of the revised text has been included for clarity (see highlighted text in* ***lines 141, 152, 164****). New supplementary table (****S1 Table****) lists all the animals.*

2c. Figure 2 & 7A: How LOD of 1 copy per mL was achieved for plasma and amniotic fluid samples? What’s the real LOD?

- ***Response****: PCR uses a plasmid standard for absolute quantification. Limit of detection is up to one RhCMV DNA copy in PCR reaction.* ***Lines 488-489*** *clarify the LOD of the PCR assay.*

2d. Figure 3: Please explain the rationale that only 3 out of 5 NHPs are described in this assessment?

- ***Response****: Samples at the early time-points were only available on three animals that were reinfected in with FL-RhCMV-gag and UCD52.*

2e. Figure 4 legend described that 4 out of NHPs were infected by UCD52+ FL-RhCMVΔRh13.1/SIVgag, inconsistent from the text.

- ***Response****: We apologize for the error. The figure legend has been corrected (****lines 778-780****) to indicate that of the 4 NHPs, 3 were infected by UCD52 and FL-RhCMV-gag and one was infected with 180.92.*

2f. Figure 5A: The same CD4 T cell count/uL data was presented as Figure 1B. However, the data are very different between the two figures.

- ***Response****: We have double-checked that the CD4 T cells/uL data in the two figures is correct and identical.*

2j. Figure 5A ELISA data: 274-05 does not have a 0.8 log increase of ED50 as labeled.

- ***Response:*** *The colors for log increase and CD4 T cells had been interchanged by mistake. This error has been corrected in revised Figure 5A.*

2h. Figure 6A: Please include the same data for JP01 and especially KK24 which had the cCMV case.

- ***Response****: SIV gag DNA was not detected in JP01 and KK24. Supplementary* ***figure S4A*** *has been added to show the SIV gag PCR data in all three animals including the negative data.*

3. Figure 3: please explain the drop of %B cells upon CD4 T cell depletion

- ***Response****: The mechanism of transient B cell decline after anti-CD4 antibody infusion and reinfection is not known. Its transient nature suggests the possibility of sequestration. Transient B cell decline has also been reported in acute SIV infection (Figure 2 in Kuhrt et al, J Virol 2010;* [*https://journals.asm.org/doi/epub/10.1128/jvi.01966-09*](https://journals.asm.org/doi/epub/10.1128/jvi.01966-09)*) due to early depletion of naïve and memory B cell subsets followed by partial recovery of memory B cells. In this study we did not evaluate the different B cell subsets.*

4. Table 1: It will be nice to include to table 1 or a supplemental table about dates of depletion/challenge, center/location, virus stock#/dose (same virus stock used for all studies?), so the reader can appreciate the potential differences among studies and animals.

- ***Response****: A supplementary table (****S1 Table****) has been added with the requested details. The 180.92 virus stock was the same across all the studies. The UCD52 virus stock originally obtained from Dr Peter Barry and used for the Bialas PNAS 2015 and Nelson JCI Insight 2017 studies, was expanded and re-titered in the Permar lab for the reinfection studies.*
